# Are patients self-referring to Lewisham Community Wellbeing (LCW) when advised to do so by the assessment and liaison psychiatry team?

**DOI:** 10.1192/bjo.2021.219

**Published:** 2021-06-18

**Authors:** Gautam Bhatia, Thileepan Thevarajan, Jadesh Manivannan, Danny Majidian

**Affiliations:** 1 South London and Maudsley NHS Foundation Trust; 2 medical student, Kings College London

## Abstract

**Aims:**

It is well-recognised by the RCPsych that mental illness is both a cause and consequence of social exclusion, and thus social inclusion is an important part of recovery and leads to better outcomes for patients.

The Lewisham Assessment and Liaison team Neighbourhood 4 (A&L N4) is a CMHT service that acts as an intake team for all referrals into secondary care mental health services, with the purpose of assessment and brief intervention. Currently, if a patient is assessed to potentially benefit from our local social inclusion service, Lewisham Community Wellbeing (LCW), they are advised to self-refer. However, there is no follow-up as to whether patients go on to do this.

Therefore, this audit aimed to calculate:

How many patients are advised to self-refer to LCW (advised referral)

How many of these patients make the self-referral to LCW (completed referral)

**Method:**

The electronic notes for patients who were accepted by the A&L N4 team from July to September 2020 were retrospectively analysed to see if an LCW self-referral was advised. A list of these patients was then given to LCW to check whether they had self-referred.

**Result:**

A&L N4 worked with 82 patients during the study period. 16 patients were advised to self-refer to LCW- an advised referral rate of 19.5%. There was notable month-to-month variation in the advised referral rate- 29.6% in July vs. 9.4% in September.

Of the 16 patients advised to self-refer to LCW, 5 did so- a completed referral rate of 31.3%.

**Conclusion:**

The completed referral rate of 31.3% is difficult to interpret given there are no standards in this area. On one hand, the self-referral process as it currently exists is functioning; on the other, some two-thirds of patients are not making the most of a service deemed to be of benefit to their recovery.

To improve completed referral rates, efforts should be made to better ‘sell’ LCW to the patient. Potential ways of doing this would be through closer working with LCW- for example, LCW could join the clinical meetings more regularly to discuss new services they offer and feedback any patients A&L has referred. There should also be emphasis on making the self-referral process as straightforward as possible.

A secondary finding was the notable monthly variation in advised referral rates. It is important to ensure the A&L team are consistently identifying the right patients for LCW, and again, closer liaison with LCW would help achieve this.

